# Characteristics and resource utilization of high-cost users in the intensive care unit: a population-based cohort study

**DOI:** 10.1186/s12913-021-07318-y

**Published:** 2021-12-06

**Authors:** Claudia Dziegielewski, Robert Talarico, Haris Imsirovic, Danial Qureshi, Yasmeen Choudhri, Peter Tanuseputro, Laura H. Thompson, Kwadwo Kyeremanteng

**Affiliations:** 1 Department of Medicine, University of Ottawa, Ontario Ottawa, Canada; 2 ICES, University of Ottawa, Ottawa, Ontario Canada; 3 Bruyere Research Institute, Ottawa, Ontario Canada; 4 Clinical Epidemiology Program, Ottawa Hospital Research Institute, Ottawa, Ontario Canada; 5 Department of Life Sciences, Queen’s University, Kingston, Ontario Canada; 6 Division of Palliative Care, Department of Medicine, University of Ottawa, Ottawa, Ontario Canada; 7 Division of Critical Care, Department of Medicine, University of Ottawa, Ottawa, Ontario Canada

**Keywords:** Intensive care unit, High-cost users, Costs, Healthcare expenditure, Critically ill

## Abstract

**Background:**

Healthcare expenditure within the intensive care unit (ICU) is costly. A cost reduction strategy may be to target patients accounting for a disproportionate amount of healthcare spending, or high-cost users. This study aims to describe high-cost users in the ICU, including health outcomes and cost patterns.

**Methods:**

We conducted a population-based retrospective cohort study of patients with ICU admissions in Ontario from 2011 to 2018. Patients with total healthcare costs in the year following ICU admission (including the admission itself) in the upper 10th percentile were defined as high-cost users. We compared characteristics and outcomes including length of stay, mortality, disposition, and costs between groups.

**Results:**

Among 370,061 patients included, 37,006 were high-cost users. High-cost users were 64.2 years old, 58.3% male, and had more comorbidities (41.2% had ≥3) when likened to non-high cost users (66.1 years old, 57.2% male, 27.9% had ≥3 comorbidities). ICU length of stay was four times greater for high-cost users compared to non-high cost users (22.4 days, 95% confidence interval [CI] 22.0–22.7 days vs. 5.56 days, 95% CI 5.54–5.57 days). High-cost users had lower in-hospital mortality (10.0% vs.14.2%), but increased dispositioning outside of home (77.4% vs. 42.2%) compared to non-high-cost users. Total healthcare costs were five-fold higher for high-cost users ($238,231, 95% CI $237,020–$239,442) compared to non-high-cost users ($45,155, 95% CI $45,046–$45,264). High-cost users accounted for 37.0% of total healthcare costs.

**Conclusion:**

High-cost users have increased length of stay, lower in-hospital mortality, and higher total healthcare costs when compared to non-high-cost users. Further studies into cost patterns and predictors of high-cost users are necessary to identify methods of decreasing healthcare expenditure.

**Supplementary Information:**

The online version contains supplementary material available at 10.1186/s12913-021-07318-y.

## Background

Canadian health care expenditure is costly. It is expected to reach $264 billion in 2019, which translates to 11.6% of the gross domestic product (GDP) [1]. This is a particular concern within intensive care units (ICUs). The average daily cost for an ICU bed in Canada is $3592, which is threefold greater than the cost of a ward bed [2]. With the aging population and advances in medical care, ICU costs are projected to significantly rise over time, increasing by over 80% by 2026 [2–4]. This escalating demand for critical care emphasizes the need to identify cost-reducing strategies to ensure sustainability of the Canadian health care system.

A proposed intervention is to reduce spending on patients who account for a disproportionately large amount of health care spending, or high-cost users. This population has been well-described in the literature, where the top 5–10% of users consume up to 65% of hospital and nursing home costs [5–12]. In Canada, this translated to approximately $56 billion in 2016–2017 [13]. Within the ICU, the top 10% of users account for nearly 50% of the costs [14, 15]. In Canada, these patients were found to be younger, admitted with subarachnoid hemorrhage, acute respiratory failure, or complications of procedures, in contrast to high-cost users outside of the ICU, which have been found to be older with multiple medical comorbidities [6, 14, 16–19]. While several of these studies included a cost analysis, they largely limited their scope to inpatient costs in tertiary care centres [9, 14, 16, 18]. This prevents generalizability to different clinical settings, and excludes outpatient data from analysis. In this study, we described high-cost users in the ICU on a provincial level by conducting a population-based retrospective cohort study of Ontario residents. We evaluated health outcomes and analyzed cost patterns of high-cost users beyond the inpatient setting, and identified predictors of becoming a high-cost user. This information can provide insight into the characteristics of high-cost users and guide future work that can identify interventions that may reduce future healthcare expenditure.

## Methods

### Study design and population

**Fig. 1:**
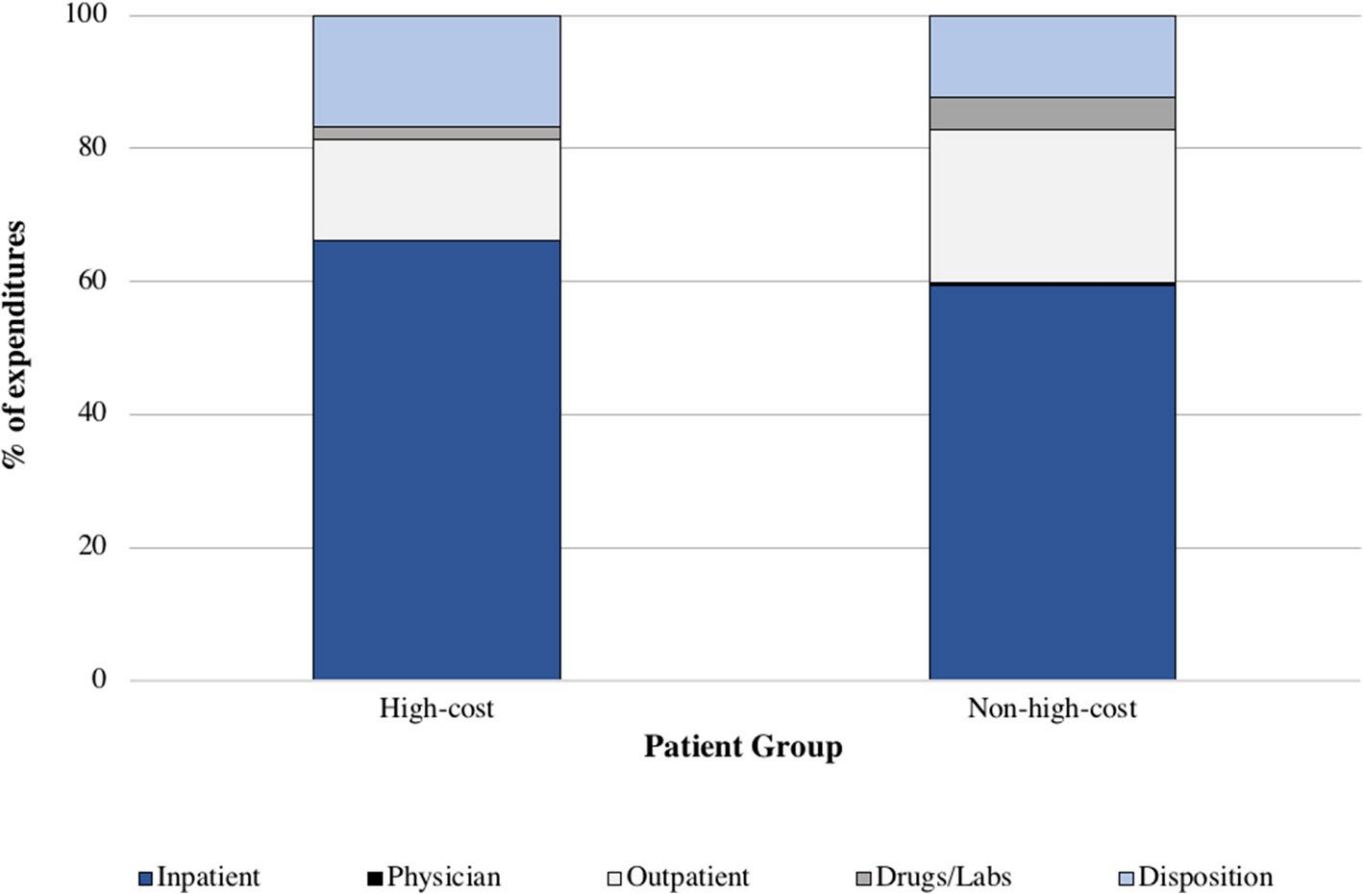
Cost proportions for high-cost and non-high-cost users admitted to the ICU. **Notes**: Costs are represented as percentages of mean total costs. Inpatient costs include mean inpatient hospital, inpatient mental health, and ED (emergency department) costs. Physician costs are mean capitation costs. Outpatient costs include all mean outpatient dialysis, outpatient cancer, same day surgery, hospital outpatient clinic, total FFS and non-FFS visits, and OHIP non-physician costs. FFS = fee-for-service. Drugs/labs include mean OHIP lab and ODB drug costs. Disposition costs include mean complex continuing care, long term care, rehab, and home care services costs

We conducted a retrospective observational population-based cohort study in Ontario, Canada. The study population included all patients aged 18 years or older with at least one admission to an ICU from January 1, 2011 to March 31, 2018. The first ICU admission was used as the index admission. For patients with multiple ICU admissions within this timeframe, only the first ICU admission was included. Transfers to different hospitals were included in the same episode of care. Follow up data was obtained up to March 31, 2019. Patients were excluded if the date of admission or date of discharge were missing, the ICU LOS was < 48 h, or if they were not OHIP (Ontario Health Insurance Plan) eligible during ICU admission or follow up (Additional Files Fig. 1).

### Data sources and outcome variables

We conducted a population-based, retrospective cohort study using health administrative databases in Ontario, Canada. In Ontario’s single-payer healthcare system, all medically necessary health care services, physician, hospital, and demographic information for residents are recorded in these databases. Databases were linked and then anonymized at the individual level at the Institute for Clinical Evaluative Sciences (ICES), a non-profit custodian of provincial health data. ICES is funded by an annual grant from the Ontario Ministry of Health and Long-term Care. Patient demographics and deaths were obtained using the Registered Persons Database. Acute care hospitalizations, including information on outcome variables, were captured using the Discharge Abstract Database and Ontario Mental Health Reporting System. Comorbid conditions were presented using the Charlson comorbidity score, a score calculated based on a list of medical conditions a patient has within hospital records [20]. We identified complex chronic diseases among our cohort, using previously described methods (Additional Files Table 1) [21]. All other conditions were based on the presence of any one inpatient hospital diagnostic code, or two or more outpatient physician billing codes within a 2-year period, using relevant ICD, Version 9 (ICD-9) and ICD-10 codes. The National Ambulatory Care Reporting System was used to obtain information on emergency department (ED) visits. The OHIP Claims Database extracted data on physician fee-for-service claims for inpatient and outpatient services. The Ontario Drug Benefit Claims database tracked data on prescription medications dispensed to patients aged 65 years or older. The National Rehabilitation Reporting System was used for inpatient rehabilitation programs, the Continuing Care Reporting System for data on long-term care (i.e., nursing home) and complex continuing care use, and Home Care databased for data on home care use. Codes can be found in Additional Files Tables 1, 2 and 3.

We obtained the total and sector-specific direct healthcare costs accumulated in the year following the date of the index ICU admission (including the admission itself). These were records of healthcare paid for by the Ontario Ministry of Health and Long-term Care (MOHLTC). We estimated the costs associated with each record by multiplying ICU length of stay by an average daily ICU cost per patient, using previously described standardized costing guidelines [22]. Briefly, we’ve taken a payer (MOHLTC) costing perspective, using person-level health care expenditures that accounts for data for health care utilization and cost information per use. Cost information for sectors (e.g., hospitals, complex continuing care, rehab) that have global budgets (e.g., by institution or by health region) were determined using a top-down approach through case-mix methodology. Sectors that have fee payments associated with each use (e.g., drug cost, or cost paid out to physician) had costs estimated directly. We expressed all costs in 2018 Canadian dollars, and past costs were adjusted for inflation using the yearly Consumer Price Index reported by Statistics Canada [23].

We derived outcome variables such as ICU and hospital LOS, inpatient procedures/interventions, and mortality. Patients were also followed up for up to 1 year post-high-cost admission to determine if there were recurrent ED visits, or re-admissions to hospital or ICU. We determined discharge disposition using a hierarchy approach (Additional Files Table 4).

We examined predictors of becoming a high-cost user, using variables that preceded index ICU admission. These included age, sex, income quintile, comorbidities, and ED and hospital visits prior to index ICU admission.

### Patient groups

We separated patients into 1) patients with total healthcare costs in the upper 10th percentile, from index ICU admission to 1-year follow-up, or “high-cost” users, and 2) patients in the remaining 90%, or “non-high-cost” users. We chose to represent high-cost users as the upper 10th percentile based on previous studies using the same strategy [14, 15, 18].

### Statistical analysis

We conducted statistical analysis using SAS Enterprise Guide 7.1 (SAS Institute Inc., Cary, NC, USA). We presented descriptive statistics as percentages or mean (with confidence intervals), as appropriate. We included standardized differences. We used a logistic regression to model the dichotomous outcome variable (whether an individual is a high- or non-high-cost user). The predictor variables of interest were age, sex, income quintile, Charlson score, number of ED and hospital visits before the index admission, as well as presence of the most prevalent comorbidities. *P*-values of < 0.05 were considered statistically significant.

## Results

We identified a total of 370,061 patients admitted to the ICU based on inclusion criteria. Of these, 37,006 (10.0%) were classified as high-cost users and 333,055 (90.0%) represented non-high-cost users. Baseline patient characteristics for both groups are described in Table 1. High-cost users had a mean age of 64.2 years (95% confidence interval [CI] 64.0–64.3 years), compared to 66.1 years (95% CI 66.1–66.2 years) in the non-high-cost user group. There were 21,586 (58.3%) males in the high-cost group compared to 190,402 (57.2%) males in the non-high-cost group. Nearly 90% of high-cost users lived in urban areas, while 84.5% of non-high-cost users lived in urban areas. There were high rates of hypertension (52.8% vs. 48.9%), diabetes (38.2% vs. 30.6%), and cancer (30.9% vs. 29.8%) in the high-cost user group. The Charlson comorbidity index score was ≥3 in 41.2% of high-cost users compared to 27.9% in non-high-cost users.

**Table 1:** Baseline patient characteristics of high-cost users and non-high-cost users admitted to the ICU

Variable	High-cost (***n*** = 37,006)	Non-high-cost (***n*** = 333,055)	Standardized Difference
Age, mean (95% CI)			
Age categories, n (%)	64.2 (64.0–64.3)	66.1 (66.1–66.2)	0.12
18–49	6108 (16.5%)	49,482 (14.9%)	0.05
50–64	10,539 (28.5%)	88,305 (26.5%)	0.04
65–79	14,211 (38.4%)	119,934 (36.0%)	0.05
80+	6148 (16.6%)	75,334 (22.6%)	0.15
Male, n (%)	21,586 (58.3%)	190,402 (57.2%)	0.02
Neighbourhood income quintile, n (%)			
Lowest	9761 (26.4%)	80,545 (24.2%)	0.05
Low	8157 (22.0%)	71,908 (21.6%)	0.01
Middle	6945 (18.8%)	65,409 (19.6%)	0.02
High	6187 (16.7%)	59,141 (17.8%)	0.03
Highest	5783 (15.6%)	54,845 (16.5%)	0.02
Missing	173 (0.5%)	1207 (0.4%)	0.02
Rurality, n(%)			
Urban	32,944 (89.0%)	281,388 (84.5%)	0.13
Rural	3990 (10.8%)	51,218 (15.4%)	0.14
Missing	72 (0.2%)	449 (0.1%)	0.01
Charlson comorbidity index score, n (%)			
0–1	14,145 (38.2%)	180,796 (54.3%)	0.33
2	7603 (20.5%)	59,499 (17.9%)	0.07
3+	15,258 (41.2%)	92,760 (27.9%)	0.28
Chronic conditions (by diagnosis), n (%)			
Hypertension	19,527 (52.8%)	162,938 (48.9%)	0.08
Diabetes	14,145 (38.2%)	101,914 (30.6%)	0.16
Cancer	11,452 (30.9%)	99,125 (29.8%)	0.03
Osteoarthritis	10,814 (29.2%)	92,475 (27.8%)	0.03
Renal Failure	9277 (25.1%)	44,061 (13.2%)	0.30
CHF	8085 (21.8%)	59,452 (17.9%)	0.10
CAD	7041 (19.0%)	73,569 (22.1%)	0.08
Mental Health	6196 (16.7%)	43,747 (13.1%)	0.10
COPD	5372 (14.5%)	46,505 (14.0%)	0.02
Arrhythmia	4517 (12.2%)	39,370 (11.8%)	0.01
Stroke	2555 (6.9%)	14,448 (4.3%)	0.11
Asthma	2418 (6.5%)	20,378 (6.1%)	0.02
MI	1116 (3.0%)	14,499 (4.4%)	0.07
Number of hospital admissions pre-ICU admission			
0	22,576 (61.0%)	233,725 (70.2%)	0.19
1	7772 (21.0%)	63,017 (18.9%)	0.05
2+	6658 (18.0%)	36,313 (10.9%)	0.2
Number of ED visits pre-ICU admission			
0	17,151 (46.3%)	172,423 (51.8%)	0.11
1	8793 (23.8%)	77,267 (23.2%)	0.01
2+	11,062 (30.0%)	83,365 (25.0%)	0.11

Age is represented by mean years (confidence interval [CI]). The rest of the data is represented by n (%), where *n* = number of patients. Chronic conditions are considered active comorbidities within the last 2 years prior to index ICU admission

*CAD* coronary artery disease, *CKD* chronic kidney disease, *CHF* congestive heart failure, *COPD* chronic obstructive pulmonary disease, *MI* myocardial infarction

Outcome variables including LOS, procedures, mortality, and discharge disposition are described in Table 2. Mean ICU and total hospital LOS was four times greater for high-cost users when compared to non-high cost users (22.4 days, 95% CI 22.0–22.7 days; 59.9 days, 95% CI 59.1–60.6 days vs. 5.56 days, 95% CI 5.54–5.57 days; 13.8 days, 95% CI 13.8–13.9 days, respectively). Palliative care was involved more frequently for non-high-cost users (9.0% vs. 6.9%). High-cost users utilized higher rates of interventions, including invasive mechanical ventilation (63.4% vs. 29.8%), feeding tubes (17.5% vs. 3.0%), and dialysis (18.2% vs. 3.5%). High-cost users were more likely to be discharged to alternative placements other than home independently (77.4%), compared to non-high-cost users (42.2%). High-cost users had a lower mortality rate in hospital (10.0%) compared to non-high-cost users (14.2%). However, they had a higher mortality rate 1-year post discharge (27.0% vs. 25.2%). There were higher rates of ED visits both pre- (53.7% vs. 48.2%) and post-ICU admission (48.8% vs. 41.8%) for high-cost users.

**Table 2:** Outcome variables for high-cost and non-high cost users admitted to the ICU

Variables	High-cost (***n*** = 37,006)	Non-high-cost (***n*** = 333,055)	Standardized Difference
ICU Length of stay, mean (95% CI)	22.4 (22.0–22.7)	5.56 (5.54–5.57)	0.68
Acute Length of stay, mean (95% CI)	37.5 (36.9–38.1)	8.24 (8.20–8.29)	0.69
Total Length of stay, mean (95% CI)	59.9 (59.1–60.6)	13.8 (13.8–13.9)	0.89
ALC Length of stay, mean (95% CI)	12.3 (11.8–12.7)	1.00 (0.97–1.03)	0.36
Palliative care involvement, n (%)	2544 (6.9%)	30,063 (9.0%)	0.08
Procedures/interventions, n (%)			
Invasive mechanical ventilation	23,464 (63.4%)	99,222 (29.8%)	0.72
Dialysis	6727 (18.2%)	11,809 (3.5%)	0.48
Feeding tube	6467 (17.5%)	9999 (3.0%)	0.49
Bronchoscopy	4267 (11.5%)	10,023 (3.0%)	0.33
CPR	1658 (4.5%)	6291 (1.9%)	0.15
Non-invasive mechanical ventilation	1411 (3.8%)	11,671 (3.5%)	0.02
Defibrillation	1198 (3.2%)	6460 (1.9%)	0.08
PCI	1166 (3.2%)	26,888 (8.1%)	0.22
Blood transfusion	45 (0.1%)	99 (0.0%)	0.03
Discharge Disposition, n (%)			
Discharged to CCC/Rehab	15,622 (42.2%)	30,602 (9.2%)	0.82
Discharged to LTC	1589 (4.3%)	7874 (2.4%)	0.11
Discharged to Home with Homecare	11,448 (30.9%)	101,982 (30.6%)	0,01
Discharged to Home without Homecare	4638 (12.5%)	145,181 (43.6%)	0.74
Death in Hospital	3709 (10.0%)	47,416 (14.2%)	0.13
Mortality, n (%)			
Death in hospital	3709 (10.0%)	47,416 (14.2%)	0.13
Death 1 year post admission	9979 (27.0%)	83,810 (25.2%)	0.04
Number of ICU re-admissions post-ICU admission			
0	21,988 (59.4%)	282,883 (84.9%)	0.59
1	10,802 (29.2%)	42,714 (12.8%)	0.41
2+	4216 (11.4%)	7458 (2.2%)	0.37
Number of hospital acute re-admissions post-ICU admission			
0	10,360 (28.0%)	201,670 (60.6%)	0.69
1	10,505 (28.4%)	81,401 (24.4%)	0.09
2+	16,141 (43.6%)	101,637 (30.5%)	0.66
Number of ED visits post-ICU admission			
0	18,950 (51.2%)	193,724 (58.2%)	0.14
1	7608 (20.6%)	65,967 (19.8%)	0.02
2+	13,928 (37.6%)	73,364 (22.0%)	0.14

Length of stay (LOS) is represented by mean days (95% confidence interval). Acute LOS is defined as hospital admission outside of the ICU (ie. ward). Total LOS is the sum total of ICU and acute LOS. A one-year lookback period before index ICU admission date is used to determine the number of hospital admissions or ED visits pre-ICU admission. A one-year follow up period after index ICU admission date is used to determine the number of ICU re-admissions, hospital re-admissions or ED visits post-ICU admission

*PCI* percutaneous coronary intervention, *CPR* cardiopulmonary resuscitation, *CCC* complex continuing care, *LTC* long term care, *ED* emergency department

Cost data from index ICU admission to 1-year follow up is summarized in Table 3, and represented as mean costs per average patient. Inpatient hospital costs were significantly higher for high-cost ($153,348, 95% CI $152,145–$154,552), compared to non-high-cost users ($25,620, 95% CI $25,547–$25,693). Inpatient costs accounted for the highest proportion of costs, more than 50% for both high-cost and non-high-cost users (Fig. 1). Costs for alternative disposition placements including complex continuing care and rehab were significantly higher for high-cost ($20,154, 95% CI $19,679–$20,628; $11,127, 95% CI $10,895–$11,358) vs. non-high-cost users ($809, 95% CI $791–$827; $1716, 95% CI $1693–$1740). Finally, mean total healthcare costs were substantially increased for high-cost users ($238,231, 95% CI $237,020–$239,442) compared to non-high-cost users ($45,155, 95% CI $45,046–$45,264). High-cost users accounted for 37.0% of total healthcare costs, equivalent to approximately $8.82 billion in total healthcare expenditure.

**Table 3:** Cost breakdown for high-cost and non-high-cost users admitted to the ICU

Variables	High-cost (***n*** = 37,006)	Non-high Cost (***n*** = 333,055)	Standardized Difference
Inpatient Hospital Costs	$153,348 ($152,145–$154,552)	$25,620 ($25,547–$25,693)	1.50
Inpatient Mental Health Costs	$2473 ($2265–$2682)	$285 ($274–$296)	0.15
ED Costs	$1625 ($1604–$1646)	$916 ($912–$920)	0.42
Outpatient Dialysis Costs	$7585 ($7352–$7818)	$397 ($379–$414)	0.43
Outpatient Cancer Costs	$1613 ($1527–$1699)	$774 ($759–$789)	0.12
Same Day Surgery Costs	$662 ($639–$686)	$676 ($669–$683)	0.01
CCC Costs	$20,154 ($19,679–$20,628)	$809 ($791–$827)	0.58
LTC Costs	$2607 ($2517–$2698)	$1057 ($1035–$1078)	0.20
Rehab Costs	$11,127 ($10,895–$11,358)	$1716 ($1693–$1740)	0.56
Home Care Services Costs	$6016 ($5883–$6150)	$2016 ($1999–$2033)	0.40
Hospital Outpatient Clinic Costs	$3662 ($3628–$3696)	$1516 ($1511–$1522)	0.81
Total FFS Visits	$22,176 ($22,019–$22,334)	$6736 ($6721–$6752)	1.35
Non-FFS ED Visits	$7.91 ($7.49–$8.33)	$4.64 ($4.55–$4.72)	0.10
Non-FFS GP Visits	$10.7 ($10.4–$11.0)	$13.7 ($13.6–$13.8)	0.11
Non-FFS Medical Oncology Visits	$105 ($97–$112)	$48.2 ($46.9–$49.5)	0.10
Non-FFS Radiation Oncology Visits	$27.3 ($25.9–$28.6)	$15.6 ($15.3–$15.9)	0.11
Other Non-FFS Visits	$487 ($471–$503)	$220 ($218–$222)	0.23
OHIP Non-Physician Costs	$59.2 ($56.6–$61.8)	$43.1 ($42.4–$43.9)	0.07
OHIP Lab Costs	$179 ($176–$182)	$175 ($174–$176)	0.01
ODB Drug Costs	$4156 ($3984–$4328)	$1952 ($1937–$1966)	0.18
Physician Capitation Costs	$151 ($149–$153)	$165 ($164–$165)	0.08
Total Healthcare Costs	$238,231 ($237,020–$239,442)	$45,155 ($45,046–$45,264)	2.22

All costs are represented as mean (95% confidence interval). Costs are accumulated in the year following the date of the index ICU admission (including the admission itself). All costs are expressed in CDN ($), adjusted to 2018 prices

*ED* emergency department, *OHIP* Ontario Health Insurance Plan, *ODB* Ontario Drug Benefit, *CCC* complex continuing care, *LTC* long term care, *FFS* fee-for-service, *GP* general practitioner

**Fig. 2:**
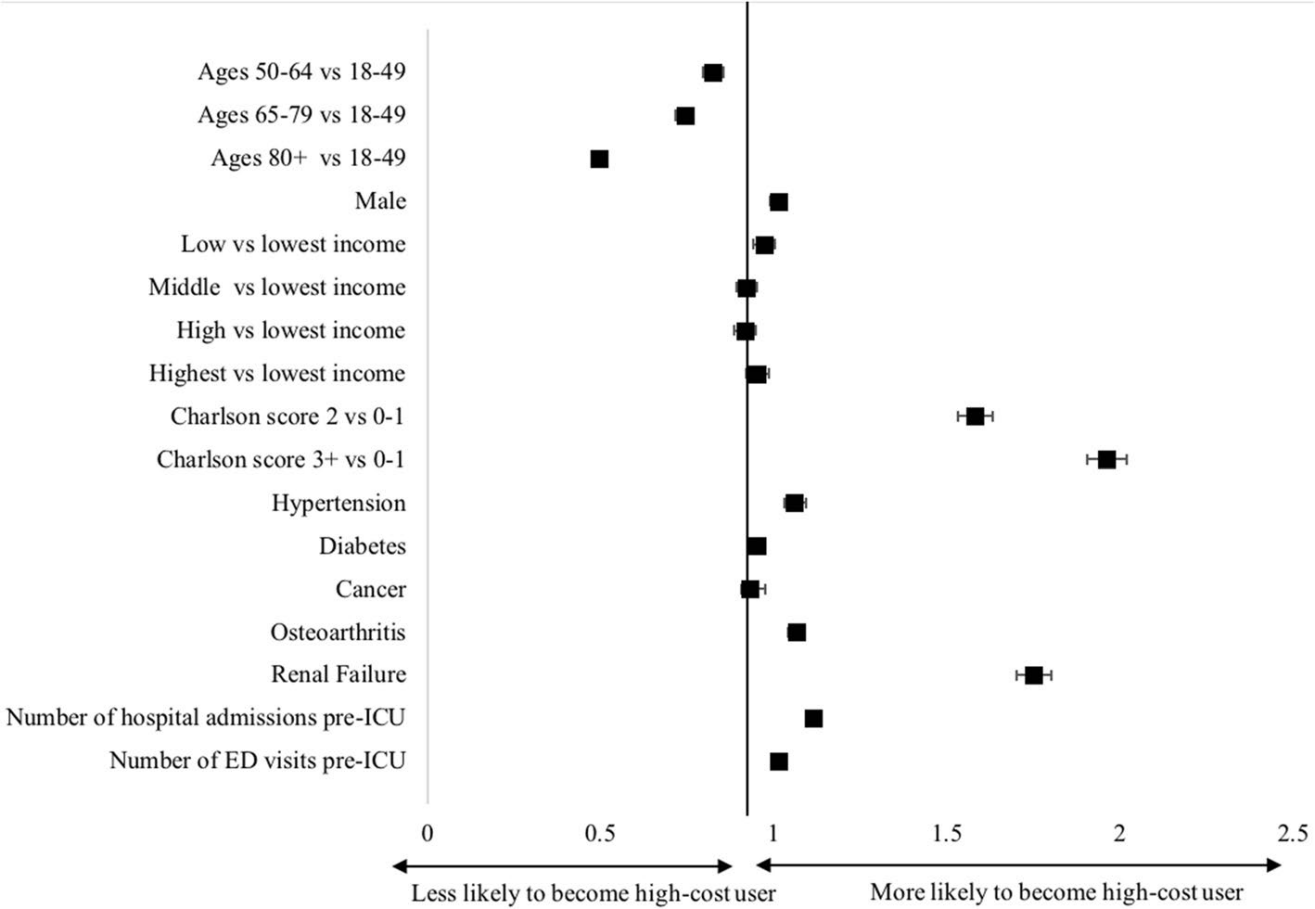
Forest plot displaying odds ratios of becoming a high-cost user in the ICU. Notes: Odds ratios are represented by squares. Horizontal error bars represent 95% confidence intervals. Age 18–49, lowest income quintile, and Charlson comorbidity score of 0–1 were used as the reference comparison group. CAD = coronary artery disease; ED = emergency department

Multivariate logistic regression analysis was performed (Fig. 2, Additional Files Table 5). Patients aged 80 and older (OR 0.50, 95% CI 0.48–0.52) were less likely to be high-cost users, compared to those under 50 years of age. Those with more than 1 (OR 1.58, 95% CI 1.53–1.63) or 2 comorbidities were very likely to be in the high-cost user group (OR 1.96, 95% CI 1.91–2.02). Of the top 5 comorbidities identified in this patient cohort, having a past medical history including renal failure (OR 1.75, 95% CI 1.70–1.80), osteoarthritis (OR 1.07, 95% CI 1.04–1.09), and hypertension (OR 1.06, 95% CI 1.03–1.08) were associated with high-cost user status. Both hospital admissions (OR 1.11, 95% CI 1.10–1.13) and ED visits (OR 1.02, 95% CI 1.01–1.02) prior to ICU admission were slightly associated with high-cost user status.

## Discussion

In this retrospective population-based cohort study, we describe the characteristics, outcomes, and cost patterns of high-cost users in the ICU that account for the upper 10th percentile of total costs to the healthcare system in Ontario, Canada. To our knowledge, there have been no prior studies describing high-cost users in the ICU on a provincial level and across multiple healthcare sectors. We found that high-cost users are younger, male, and come from lower income families. They have more comorbidities compared to non-high-cost users. They have a longer LOS in the ICU and hospital, lower in-hospital mortality rates, but higher rates of being discharged to placements other than to home independently. They were more frequently readmitted to ICU and acute care. High-cost users had a five-fold increase in total costs compared to non-high-cost users and despite only representing 10% of the cohort, accounted for more than one-third of total healthcare costs, which translated to $8.82 billion in total.

LOS has been previously well-described to be a significant driver of increased costs in the ICU [24–26]. This is supported by our study, which shows both ICU and total hospital LOS is four-fold greater for high-cost users. Furthermore, high-cost users have higher rates of interventions during their ICU admission. Invasive mechanical ventilation and dialysis are both costly procedures that often require ICU stay, and thus prolong LOS for patients in critical care [27, 28]. Taken together, the increased interventions and LOS likely contribute to significantly increased costs during admission. This correlates to our finding that inpatient costs contribute to more than 50% of total costs accrued by high-cost users. The second highest proportion of costs was driven by dispositioning, as more than 75% of high-cost users were discharged to a placement other than to home independently. Patients who are critically ill often require longer to recover and become frailer, requiring costly dispositioning for increased supports [29–31]. They often stay in hospital while awaiting placement, which prolongs LOS, increases costs, and may even contribute to further deterioration in health [32–34]. This highlights the need to increase the number of community supports to improve flow in the hospital and reduce unnecessary acute care stay. High-cost users had lower mortality during hospital admission than non-high-cost users, which has been previously demonstrated [14]. As costs appear to be significantly impacted by LOS, those with severe illnesses may have died sooner, reducing their contribution to overall costs. Although high-cost users had lower mortality during hospital stay, they had higher mortality at follow-up, which may be related to increased morbidity suggested by higher comorbidity scores and increased alternative disposition placements. Surprisingly, palliative care was less frequently involved in the care of high-cost users, although these patients appear to be more ill, comorbid, and frail and have higher out-of-hospital mortality. This highlights the importance of addressing goals of care early and re-evaluating which patients will benefit from critical care and aggressive interventions.

This study has identified several predictors of becoming a high-cost user. These include younger age, lower income, increased comorbidities, and prior hospital visits. Previous studies have showed conflicting reports on average age of high-cost users [14, 18, 35–37]. In our study, older patients, especially those age 80 and over, are less likely to be high-cost users. This is likely due to increased frailty and multi-morbidity predisposing them to earlier mortality and thus less likely to be in acute care long enough to accrue high costs [38, 39]. Socioeconomic status has been previously shown to be a significant predictor of becoming a high-cost user [37]. While preventing low income status is challenging, modifying health behaviours may help reduce medical illness that may predispose a patient to becoming a high-cost user. As expected, patients with more comorbidities are more likely to become high-cost users, as supported in previous studies [36, 40]. Common comorbidities such as renal failure and hypertension were associated with becoming a high-cost user, as patients are often frail, and develop complications of these conditions with lower mortality that may require critical care services [40, 41]. ED visits and hospital admissions prior to ICU admission were also associated with becoming a high-cost user, suggesting these patients are frequent users of the healthcare system. This highlights the importance of preventative care and having a primary care physician or specialist to closely follow and manage patients with several comorbidities.

Further studies should explore high costs in the ICU, and strategies of preventing patients from becoming high-cost users. This may include prospective trials that examine early palliative care approaches and multidisciplinary programs that integrate care. Palliative care has been shown to reduce ICU LOS and costs [42, 43]. Communication with patients and families on goals of care results in less frequent pursuit of ICU-level care while enhancing quality of care [44]. Furthermore, interdisciplinary care with several healthcare providers may help medically optimize patients and prevent hospital admissions, although literature is not available in the ICU setting [45–47]. While high-cost users are well-described in the literature, it is unclear how much of these costs are preventable. Some studies suggest that in the inpatient setting, less than 20% of costs may be preventable, but these were mostly limited to preventable ED visits and hospital re-admissions, which may not apply to several ICU admissions [37, 48]. This should be the focus of future studies, which could inform strategies in reducing costs while optimizing quality of care for patients in the ICU setting.

While this study involves a large population cohort that is robust and generalizable, there are several limitations. Firstly, we utilized health administrative data, which lacks certain detailed clinical variables and therefore limits the analysis of predictors of high-cost users. Admission diagnoses were not available in the database, and therefore information about which diagnoses are associated with higher costs could not be obtained. Functional data was not obtainable, which limits our understanding of the functional status of patients who become high-cost users. However, the higher proportion of high-cost users who are discharged to placements other than home without supports suggests decreased functional independence. While total costs were available, daily costs could not be obtained, which is likely variable depending on interventions and day of ICU stay. ODB captures drug costs for residents aged 65 years and older, which likely underestimates these costs; however, these costs are factored into the costing algorithm since hospital costs are top down. Cost analysis provided large category breakdown of costs; however, details such as specialist outpatient visits, or costs of certain interventions such as invasive mechanical ventilation, were not available. This information could be helpful to determine if patients being discharged from ICU receive appropriate follow up that may prevent future readmissions, or if certain interventions drive up costs [49]. However, the fact that there are less patients discharged home without care in the high-cost user group suggests a degree of functional impairment. Finally, the retrospective nature of this study allows association, but not causation, to be determined.

## Conclusion

High-cost ICU patients in Ontario, Canada are younger, male, with increased comorbidities and lower mortality, and account for nearly 40 % of total healthcare costs. Drivers of increased costs include LOS, increased interventions, and increased need for alternative disposition placement. Further research is necessary in identifying modifiable factors of becoming a high-cost user, and effective methods of preventing patients from becoming high-cost users, such as increased community supports, early palliative care, and interdisciplinary health teams. Together, these strategies may help reduce overall healthcare expenditure while improving patient outcomes.

## Supplementary Information

**Additional file 1.**

## Data Availability

All data generated or analyzed during this study are included in this published article [and its supplementary file]. The dataset from this study is held securely in coded form at ICES. While data sharing agreements prohibit ICES from making the dataset publicly available, access may be granted to those who meet pre-specified criteria for confidential access, available at www.ices.on.ca/DAS. The full dataset creation plan and underlying analytic code are available from the authors upon request, understanding that the computer programs may rely upon coding templates or macros that are unique to ICES and are therefore either inaccessible or may require modification.
