# Supplementary material for: Characteristics and resource utilization of high-cost users in the intensive care unit: a population-based cohort study
Source: BMC Health Serv Res. 2021 Dec 6;21:1312. doi: 10.1186/s12913-021-07318-y (PMC8647444; doi:10.1186/s12913-021-07318-y)
Supplement: Supplementary file 1 — Additional file 1. [file 12913_2021_7318_MOESM1_ESM.docx]

**Additional Files**

**Characteristics and Resource Utilization of High-Cost Users in the Intensive Care Unit: A Population-based Cohort Study**

Claudia Dziegielewski^1^, Robert Talarico^3^, Haris Imsirovic^3^, Danial Qureshi^3,4,6^, Yasmeen Choudhri, Peter Tanuseputro^3,4,5,6^, Laura H. Thompson^3^, Kwadwo Kyeremanteng^2,5^

^1^Department of Medicine, University of Ottawa, Ottawa, Ontario, Canada

^2^Division of Critical Care, Department of Medicine, University of Ottawa, Ottawa, Ontario, Canada

^3^ICES, University of Ottawa, Ottawa, Ontario, Canada

^4^Bruyere Research Institute, Ottawa, ON, Canada

^5^Division of Palliative Care, Department of Medicine, University of Ottawa, Ottawa, Ontario, Canada

^6^Clinical Epidemiology Program, Ottawa Hospital Research Institute, Ottawa, ON, Canada

^7^Queen’s University, Ontario, Canada

Corresponding author: Claudia Dziegielewski, Department of Medicine, University of Ottawa, 451 Smyth Rd, Ottawa, ON, Canada, K1H 8M5. Email: cldziegielewski@toh.ca.

**Additional File Figure 1**: Cohort Creation Flow Chart.

All ICU admission records with valid IKN (ICES Key Number)

*n*=1,062,617

Exclude records with missing date of admission/discharge

*n*=7

Exclude records with multiple ICU admissions per episode of care

*n*=75,958

First ICU admission per episode of care

*n*=986,652

*n*=1,062,617

Exclude admissions with ICU LOS <48 hours

*n*=438,634

Exclude patients aged <18 and >105

*n*=95,133

n=438634

Exclude patients not OHIP eligible consecutively from first ICU admission until 1 year after admission

*n*=0

n=438634

Exclude multiple episodes of care per patient

*n*=82,824

Patient inclusion list

*n*=370,061

*n*=1,062,617

Non-high-cost users

*n*=333,055

High-cost users

*n*=37,006

**Additional File Table 1**: Ontario Health Insurance Plan (OHIP), International Classification of Diseases, Version 9 (ICD-9), and Version 10 (ICD-10) diagnostic codes for categorization of comorbidities.

| **Condition [reference for validated algorithm]** | **ICD 9 / OHIP** | **ICD 10** | **ODB*** |
| --- | --- | --- | --- |
| Acute Myocardial Infarction (AMI) [1] | 410 | I21, I22 |  |
| Osteo- and other Arthritis:   1. Osteoarthritis 2. Other Arthritis (includes Synovitis, Fibrositis, Connective tissue disorders, Ankylosing spondylitis, Gout Traumatic arthritis, pyogenic arthritis, Joint derangement, Dupuytren’s contracture, Other MSK disorders) | 715  727, 729, 710, 720, 274, 716, 711,718, 728, 739 | M15-M19  M00-M03, M07, M10, M11-M14, M20-M25, M30-M36,  M65-M79 |  |
| Arthritis - Rheumatoid arthritis [2] | 714 | M05-M06 |  |
| Asthma [3] | 493 | J45 |  |
| Cancer | 140-239 | C00-C26, C30-C44, C45-C97 |  |
| Cardiac Arrhythmia | 427 (OHIP) / 427.3 (DAD) | I48.0, I48.1 |  |
| Congestive Heart Failure [4] | 428 | I500, I501, I509 |  |
| Chronic Obstructive Pulmonary Disease [5] | 491, 492, 496 | J41, J43, J44 |  |
| Coronary syndrome (excluding AMI) | 411-414 | I20, I22-I25 |  |
| Dementia [6] | 290, 331 (OHIP) / 046.1, 290.0, 290.1,  290.2, 290.3, 290.4, 294, 331.0, 331.1, 331.5, F331.82 (DAD) | F00, F01, F02, F03, G30 | Cholinesterase  Inhibitors |
| Diabetes [7] | 250 | E08-E13 |  |
| Hypertension [8] | 401, 402, 403, 404, 405 | I10, I11, I12, I13, I15 |  |
| (Other) Mental Illnesses | 291, 292, 295, 297, 298, 299, 301, 302, 303, 304, 305, 306, 307, 313, 314, 315, 319 | F04, F050, F058, F059, F060, F061, F062, F063, F064, F07, F08, F10, F11, F12, F13, F14, F15, F16, F17, F18, F19, F20,  F21, F22, F23, F24, F25, F26, F27, F28, F29, F340, F35, F36,  F37, F430, F439, F453, F454, F458, F46, F47, F49, F50, F51,  F52, F531, F538, F539, F54, F55, F56, F57, F58, F59, F60,  F61, F62, F63, F64, F65, F66, F67, F681, F688, F69, F70,  F71, F72, F73, F74, F75, F76, F77, F78, F79, F80, F81, F82,  F83, F84, F85, F86, F87, F88, F89, F90, F91, F92, F931,  F932, F933, F938, F939, F94, F95, F96, F97, F98 |  |
| Mood, anxiety, depression and other nonpsychotic disorders | 296, 300, 309, 311 | F30, F31, F32, F33, F34 (excl. F34.0), F38, F39, F40, F41,  F42, F43.1, F43.2, F43.8, F44, F45.0, F45.1, F45.2, F48,  F53.0, F68.0, F93.0, F99 |  |
| Osteoporosis | 733 | M81, M82 |  |
| Renal failure | 403, 404, 584, 585, 586, v451 | N17, N18, N19, T82.4, Z49.2, Z99.2 |  |
| Stroke (excluding transient ischemic attack) | 430, 431, 432, 434, 436 | I60I64 |  |

**Notes**: All available health administrative data (OHIP, DAD, ODB) prior to index is used to ascertain disease status, with the exception of AMI (1 year prior to index), Cancer (2 years), Mood Disorder (2 years) and Other Mental Illnesses (2 years) as these conditions are considered episodic. AMI, Asthma, COPD, CHF, Dementia, Diabetes, Hypertension, IBD, and Rheumatoid Arthritis are based on validated case algorithms/ ICES cohorts (see 1-9 below, respectively). All other conditions required at least one diagnosis code recorded in acute care (DAD) or two diagnosis codes recorded in physician billings (OHIP) within a two-year period. ODB prescription drug records are not available for the majority of persons under the age of 65. DAD=Discharge Abstract Database; ICD = International Classification of Disease; ODB = Ontario Drug Benefit Claims database; OHIP = Ontario Health Insurance Plan Claims Database.

References:

1. Austin PC, Daly PA, Tu JV. A multicenter study of the coding accuracy of hospital discharge administrative data for patients admitted to cardiac care units in Ontario. Am Heart J 2002;144:290–6.

2. Widdifield J, Bernatsky S, Paterson JM, Tu K, Ng R, Thorne JC, et al. Accuracy of Canadian health administrative databases in identifying patients with rheumatoid arthritis: a validation study using the medical records of rheumatologists. Arthritis Care Res 2013; 65(10): 1582-91.

3. Gershon AS, Wang C, Guan J, Vasilevska-Ristovska J, Cicutto L, To T. Identifying patients with physician-diagnosed asthma in health administrative databases. Can Respir J 2009;16:183–8.

4. Schultz SE, Rothwell DM, Chen Z, Tu K. Identifying cases of congestive heart failure from administrative data: a validation study using primary care patient records. Chronic Dis Inj Canada 2013;33:160–6.

5. Gershon AS, Wang C, Guan J, Vasilevska-Ristovska J, Cicutto L, To T. Identifying Individuals with Physician Diagnosed COPD in Health Administrative Databases. COPD 2009;6:388–94.

6. Jaakkimainen RL, Bronskill SE, Tierney MC, Herrmann N, Green D, Young J, et al. Identification of Physician-Diagnosed Alzheimer’s Disease and Related Dementias in Population-Based Administrative Data: A Validation Study Using Family Physicians’ Electronic Medical Records. J Alzheimers Dis 2016;54(1):337–49.

7. Hux JE, Ivis F, Flintoft V, Bica A. Diabetes in Ontario: Determination of prevalence and incidence using a validated administrative data algorithm. Diabetes Care 2002;25:512–6.

8. Tu K, Campbell NR, Chen ZL, Cauch-Dudek KJ, McAlister FA. Accuracy of administrative databases in identifying patients with hypertension. Open Med 2007;1:e18–26.

9. Benchimol EI, Guttmann A, Mack DR, Nguyen GC, Marshall JK, Gregor JC, et al. Validation of international algorithms to identify adults with inflammatory bowel disease in health administrative data from Ontario, Canada, J Clin Epidemiol 2014;67(8):887-96.

**Additional File Table 2**: Procedure/Intervention Codes.

| **Procedure/Intervention** | **Associated Database and Codes** |
| --- | --- |
| Dialysis | Discharge database: DAD  Code: 1.PZ.21- |
| Invasive Mechanical Ventilation | Discharge database: DAD  Code: 1.GZ.31.CRND, 1.GZ.31.GPND, 1.GZ.31.CA- |
| Noninvasive Ventilation | Discharge database: DAD  Code: 1.GZ.31.CB-, 1.GZ.31.JA- |
| Bronchoscopy | Discharge database: DAD  Code: 2.GM.70- |
| Percutaneous Coronary Intervention (PCI) | Discharge database: DAD  Codes: 1IJ50-, 1IJ54-, 1IJ57GQ- |
| Feeding tube | Discharge database: DAD  Code: 1.NF.53- |
| Blood transfusion | Discharge database: DAD  Code: 1.LZ.19- |
| Cardiopulmonary Resuscitation (CPR) | Discharge database: DAD  Code: 1.HZ.30- |
| Defibrillation | Discharge database: DAD  Code: 1.HZ.09- |

**Additional File Table 3**: Palliative Care Codes.

| Palliative Care | Discharge database: DAD  Code: Z515, patserv = ‘58’ |
| --- | --- |

**Additional File Table 4**: Hierarchy approach for discharge disposition of those who survived to discharge.

1. **Discharged to home with homecare** (defined as: at least one homecare service within 21 days of discharge, as identified via the Home Care Database)

2. **Discharged to rehabilitation or complex continuing care** (defined as: at least one admission to complex continuing care or a rehabilitation bed, as identified via the Continuing Care Reporting System or the National Rehabilitation Reporting System, respectively)

3. **Discharged to a long-term care facility** (defined as: at least one admission to long-term care within 2 days of discharge, as identified via the Continuing Care Reporting System – Long-term Care)

4. **Died in hospital** (defined as: death date occurring on or prior to discharge date, as identified via the Discharge Abstract Database)

5. **Discharged to home without homecare** (if patient did not meet other four criteria above, then included in this group)

*In cases where a patient is eligible for more than one disposition category, the institution with the first date is selected as priority. In cases where the dates overlap, the following hierarchy is used: death > complex continuing care/rehabilitation > long-term care facility > homecare

**Additional File Table 5:** Multivariate logistic regression analysis for high-cost users in the ICU.

| **Variable** | **OR** | **Confidence Interval** | **P value** |
| --- | --- | --- | --- |
| Age  18-49  50-64  65-79  80+ | Reference  0.82  0.74  0.50 | Reference  0.80 – 0.85  0.72 – 0.77  0.48 – 0.52 | <.0001  <.0001  <.0001 |
| Sex  Female  Male | Reference  1.01 | Reference  0.99 – 1.04 | 0.26 |
| Income  Lowest  Low  Middle  High  Highest | Reference  0.97  0.92  0.92  0.95 | Reference  0.94 – 1.00  0.90 – 0.95  0.89 – 0.95  0.92 – 0.99 | 0.079  <.0001  <.0001  0.0064 |
| Charlson comorbidity score  0-1  2  3+ | Reference  1.58  1.96 | Reference  1.53– 1.63  1.91 – 2.02 | <.0001  <.0001 |
| Top 5 Comorbidities  Hypertension  Diabetes  Cancer  Osteoarthritis  Renal failure | 1.06  0.93  0.95  1.07  1.75 | 1.03 – 1.08  0.91 – 0.95  0.93 – 0.98  1.04 – 1.09  1.70 – 1.80 | <.0001  <.0001  0.01  <.0001  <.0001 |
| Number of hospital admissions pre-ICU admission  0  1+ | Reference  1.11 | Reference  1.10 – 1.13 | <.0001 |
| Number of ED visits pre-ICU admission  0  1+ | Reference  1.02 | Reference  1.01 – 1.02 | <.0001 |

**Notes**: Odds Ratios (OR) were calculated for each variable. Age 18-49, female sex, lowest income quintile, and Charlson comorbidity score of 0-1 were used as the reference comparison group. For the top 5 medical comorbidities, the reference for each comorbidity was not having that specific comorbidity. Top 5 medical comorbidities were determined by using highest prevalence of medical conditions (excluding mood and mental health) from patient demographics (Table 1 in main manuscript). 95% confidence intervals are displayed. C-statistic for the model is 0.63. ED=emergency department.
